# Pharmacological Inhibition of Indoleamine 2,3-Dioxygenase-2 and Kynurenine 3-Monooxygenase, Enzymes of the Kynurenine Pathway, Significantly Diminishes Neuropathic Pain in a Rat Model

**DOI:** 10.3389/fphar.2018.00724

**Published:** 2018-07-11

**Authors:** Ewelina Rojewska, Katarzyna Ciapała, Anna Piotrowska, Wioletta Makuch, Joanna Mika

**Affiliations:** Department of Pain Pharmacology, Institute of Pharmacology, Polish Academy of Sciences, Krakow, Poland

**Keywords:** 1-methyl-D-tryptophan (1-D-MT), UPF 648, microglia, minocycline, indoleamine 2, 3-dioxygenase (IDO2), kynurenine 3-monooxygenase (KMO)

## Abstract

Neuropathic pain caused by a primary injury or dysfunction in the peripheral or central nervous system is a tremendous therapeutic challenge. Here, we have collected the first evidence from a single study on the potential contributions to neuropathic pain development by enzymes in the kynurenine pathway [tryptophan 2,3-dioxygenase (TDO), indoleamine 2,3-dioxygenase (IDO1/2), kynurenine 3-monooxygenase (KMO); kynureninase, 3-hydroxyanthranilate-3,4-dioxygenase (HAOO)] at the spinal cord and dorsal root ganglia (DRG) levels. At the spinal cord, mRNA levels of *IDO2, KMO*, and *HAOO* were elevated as measured on day 7 after chronic constriction injury in a rat model, parallel to the C1q-positive cell activation. According to our data obtained from primary microglial cell cultures, all enzymes of the kynurenine pathway except TDO were derived from these cells; however, the activation of microglia induced stronger changes in IDO2 and KMO. Our pharmacological studies gave evidence that the repeated intraperitoneal administration of minocycline, a microglia/macrophage inhibitor, not only attenuated tactile and thermal hypersensitivity but also diminished the levels of *IDO2* and *KMO* mRNA. Our further pharmacological studies confirmed that IDO2 and KMO enzymes take part in the development of neuropathic pain, since we observed that the repeated administration of IDO2 (1-methyl-D-tryptophan) and KMO [UPF 648 – (1S,2S)-2-(3,4-dichlorobenzoyl)cyclopropanecarboxylic acid] inhibitors diminished hypersensitivity development as measured on days 2 and 7. The results of our studies show that the kynurenine pathway is an important mediator of neuropathic pain pathology in rats and indicate that IDO2 and KMO represent novel pharmacological targets for treating neuropathy.

## Introduction

The treatment of neuropathic pain remains a major challenge. Therefore, its essential mechanisms need to be elucidated. The available International Association for the Study of Pain (IASP) data suggest that one in five Europeans suffers from moderate to severe chronic pain of various origins. Due to such conditions, they are forced to apply major changes in their lives. This is a problem of extreme importance for the quality of life of today’s aging populations. The mechanism by which the development and persistence of chronic pain are initiated is still unclear. Basing on our published results (Rojewska et al., 2014a), we postulate that as a consequence of an injury the intensification of neurotoxic kynurenine pathway activity occurs. In diseases of the central nervous system, there is a change in the metabolism of tryptophan. Metabolites of the kynurenine pathway are responsible for a broad spectrum of effects, such as the endogenous regulation of neuronal excitability and immune activation. It has already been shown that the kynurenine pathway plays an important role in the pathology of neurodegenerative diseases, autoimmune diseases, pain syndromes, migraine, and multiple sclerosis (Santamaría et al., 1996; Harris et al., 1998; Guidetti et al., 2000; Kwidzinski and Bechmann, 2007; Fejes et al., 2011; Zwilling et al., 2011). Few preliminary studies concerning possible modifications of the kynurenine pathway raise great hopes for their clinical use in the future (Majláth et al., 2013). We hypothesize that the inhibition of the activity of selected kynurenine pathway enzymes is a way to protect neurons from injuries and, as a consequence, diminish neuropathic pain development. Our previous work (Rojewska et al., 2016) showed the spinal-specific increase in the expression of kynurenine 3-monooxygenase (KMO) – an enzyme of kynurenine pathway. KMO leads to the formation of anthranilic acid, 3-hydroxyanthranilic acid, and 3-hydroxykynurenine, and these successive stages lead to an increase in quinolinic acid levels, which is a selective, endogenous *N*-methyl-D-aspartate (NMDA) receptor agonist (Lugo-Huitrón et al., 2013) whose receptor contributes to neuropathy (Parsons, 2001; Obara et al., 2003). In 2013, Amaral et al. (2013) recommended UPF 648 as a potent KMO inhibitor for therapies against neurodegenerative diseases, but there is a lack of such studies involving neuropathic pain. Recently, it has also been demonstrated that the IDO inhibitor 1-methyl-D-tryptophan (1-D-MT) is therapeutically valuable in cancer (Brito et al., 2015); however, it has not been examined for neuropathy.

The involvement of the kynurenine system in the pathology of neuropathic pain is poorly understood and requires extensive research. Therefore, we measured the changes in the mRNA of kynurenine pathway enzymes [*IDO1, IDO2, TDO, KMO, KYNU*, and 3-hydroxyanthranilate-3,4-dioxygenase (*HAOO*)] in rat spinal cord and dorsal root ganglia (DRG) tissues on the 2nd, 7th, 14th, and 28th day after the chronic constriction injury (CCI) of the sciatic nerve. In addition, we determined how the inhibition of microglia activation via the administration of minocycline influences neuropathic pain-related behaviors and levels of kynurenine pathway enzymes. Moreover, the objective of our *in vitro* studies was to determine whether and which enzymes of the kynurenine pathway are derived from microglia cells. Finally, we posed a question whether the inhibitors of two enzymes (selected on the basis of biochemical studies) of the kynurenine pathway influence hypersensitivity in a rat model of neuropathic pain – UPF 648, a KMO inhibitor (Pellicciari et al., 2003) and 1-D-MT, an IDO inhibitor (Nakamura et al., 2015).

## Materials and Methods

### Animals

Male Wistar rats (250–350 g) were provided by Charles River (Germany) and housed in cages lined with sawdust under a standard 12/12-h light/dark cycle (lights on at 06:00). Rats were given free access to water and food in a room maintained at 23–25°C and 40–60% humidity with natural lighting. This study was conducted in accordance with the recommendations of the International Association for the Study of Pain (Zimmermann, 1983) and the NIH Guide for the Care and Use of Laboratory Animals and was approved by the Local Ethics Committee (permission number 1210/2015 and 333/2018).

### Intrathecal Catheter Implantation

The rats were chronically implanted with intrathecal (i.t.) catheters under sodium pentobarbital anesthesia [60 mg/kg, intraperitoneally (i.p.)] according to the methods of Yaksh and Rudy (1976). The catheters (PE 10, INTRAMEDIC, Clay Adams, Becton Dickinson and Company, Rutherford, NJ, United States) were flushed with 70% ethanol and then with water for injections prior to insertion. They were carefully introduced through the atlanto-occipital membrane to the subarachnoid space at the rostral level of the spinal cord lumbar enlargement (L4–L6) and flushed slowly with 10 μl of water for injection, and the tip was tightened. After the catheters implantation, the rats were allowed to recover for a minimum of 1 week before the actual experiment and were monitored for physical impairments. After the surgery, all rats were fed separately. Animals with visible motor deficits were excluded from the further study.

### Chronic Constriction Injury

The CCI model was developed in reference to the methods proposed by Bennett and Xie (1988), with little modification. CCI was performed 7 days after the catheter implantation. The right sciatic nerve was exposed under sodium pentobarbital anesthesia (60 mg/kg, *i.p*.). Four ligatures (4/0 silk) were made around the nerve distal to the sciatic notch at 1-mm intervals until a brief twitch in the corresponding hind limb was observed. Then, the skin was sutured, and the awakened rats were placed back in their cages. After CCI, the rats developed long-lasting mechanical and thermal hypersensitivity.

### Behavioral Tests

#### Von Frey Test

Tactile hypersensitivity in the CCI-exposed rats was measured using an automated von Frey apparatus (Dynamic Plantar Aesthesiometer, Cat. No. 37400, Ugo Basile, Italy). The animals were placed in plastic cages with a wire mesh floor 5 min before the experiment. The von Frey filament was applied in increasing values (stimuli up to 26 g) to the midplantar surface of the hind paw, and measurements were taken automatically as described previously (Makuch et al., 2013; Rojewska et al., 2015). The ipsilateral paw was tested two times in 2-min intervals, and the mean value was calculated.

#### Cold Plate Test

Thermal hypersensitivity was assessed using a cold plate apparatus (Cold/Hot Plate Analgesia Meter, No. 05044, Columbus Instruments, United States). The animals were placed on the cold plate, and the latency of lifting the hind paw was recorded. The temperature of the plate was kept at 5°C, and the cut-off latency was 30 s, as described previously (Makuch et al., 2013; Rojewska et al., 2015). In all cases, the injured paw reacted as first.

### Drugs

Minocycline hydrochloride (*MC*; 30 mg/kg; Sigma, Schnelldorf, Germany), a microglia/macrophage inhibitor, was dissolved in water for injections and administered pre-emptively by means of intraperitoneal (*i.p*.) injections 16 and 1 h before the CCI, and then twice daily for 7 days, as we described previously (Mika et al., 2007, 2009). This method of minocycline administration was used throughout the study and in the text is referred to as “repeated administration.” This administration schedule was used because systemic microglia inhibitors attenuate the activation of microglia more efficiently when the inhibitor is injected prior to injury (Raghavendra et al., 2003; Ledeboer et al., 2005; Mika et al., 2009) and repeated afterward.

1-Methyl-D-tryptophan (20 μg/5 μl, Tocris, Warszawa, Poland), an IDO2 inhibitor, was dissolved in 50% DMSO and administered pre-emptively by *i.t.* injections 16 and 1 h before the CCI, and then once daily for 7 days.

UPF 648 (20 μg/5 μl, Tocris, Warszawa, Poland), a KMO inhibitor, was dissolved in 50% DMSO and administered pre-emptively by *i.t*. injections 16 and 1 h before the CCI, and then once daily for 7 days.

### Quantitative Reverse Transcriptase Polymerase Chain Reaction (qRT-PCR) Analysis

**FIGURE 1:**
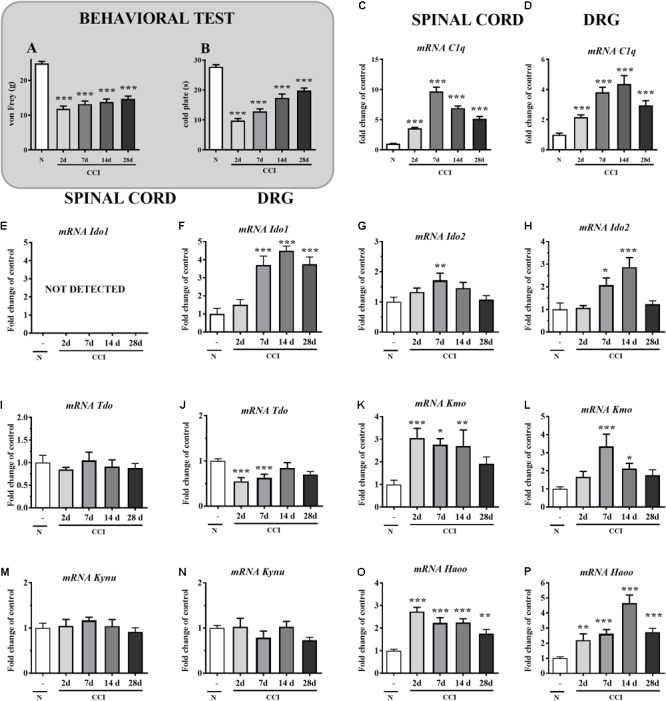
**(A,B)** Levels of mechanical (**A**; von Frey test) and thermal (**B**; cold plate test) hypersensitivity measured on the 2nd, 7th, 14th, and 28th day after the chronic constriction injury (CCI) in rats. The data are presented as the means ± SEM (10–19 rats per group). Inter-group differences were analyzed by Bonferroni’s multiple comparison tests. ^∗∗∗^*p* < 0.001 indicates a significant difference vs. naïve animals. **(C–P)** qRT-PCR analysis of the mRNA of *C1q* **(C,D)**, *IDO1* **(E,F)**, *IDO2* **(G,H)**, *TDO* **(I,J)**, *KMO* **(K,L)**, *KYNU* **(M,N)** and *HAOO* **(O,P)** in the ipsilateral dorsal spinal cord (L4–L6) and DRG (L4–L6) on days 2, 7, 14, and 28 after the CCI in rats. The data are shown as the mean fold-changes in expression relative to the control (naïve, N) levels ± SEM (4–14 samples per group). Inter-group differences were analyzed using Bonferroni’s test for multiple comparisons. ^∗^*p* < 0.05, ^∗∗^*p* < 0.01, and ^∗∗∗^*p* < 0.001 indicate significant differences compared to the naïve group.

The ipsilateral sides of the dorsal lumbar (L4–L6) spinal cord and dorsal root ganglia (DRG; L4–L6) were collected immediately after decapitation on days 2, 7, 14, and 28 after the CCI (**Figure 1**), and 7 days after the CCI (4 h after the last minocycline administration) (**Figure 3**). The tissue samples were placed in individual tubes containing the tissue storage reagent RNAlater (Qiagen Inc.) and were stored at -70°C until RNA isolation. Cell samples were collected in TRIzol reagent (Invitrogen, Carlsbad, CA, United States). Total RNA was extracted using TRIzol reagent (Invitrogen, Carlsbad, CA, United States) as described previously by Chomczynski and Sacchi (2006). The RNA concentration was measured using a NanoDrop ND-1000 Spectrophotometer (NanoDrop Technologies), and the RNA quality was determined by chip-based capillary electrophoresis using an RNA 6000 Nano LabChip Kit and an Agilent Bioanalyzer 2100 (Agilent) according to the manufacturer’s instructions. Reverse transcription was performed on 1 μg (for tissue analysis) and 500 ng (for cell culture analysis) of total RNA using Omniscript reverse transcriptase (Qiagen Inc.) at 37°C for 60 min. RT reactions were performed in the presence of an RNAse inhibitor (rRNAsin, Promega) and an oligo(dT16) primer (Qiagen Inc.). The cDNA was diluted 1:10 with H_2_O, and for each reaction, ∼50 ng of cDNA synthesized from the total RNA of an individual animal was used for the quantitative real-time PCR (qRT-PCR) reaction. The qRT-PCR was performed using Assay-On-Demand TaqMan probes according to the manufacturer’s protocol (Applied Biosystems), and the reactions were performed on an iCycler device (BioRad, Hercules). The following TaqMan primers and probes were used: Rn01527838_g1 (Hprt, rat hypoxanthine guanine phosphoribosyl transferase); Rn00570480_m1_m1 (C1qb, complement component 1, q subcomponent); Rn01482210_m1 (IDO1, indoleamine 2,3-dioxygenase 1); Rn01482543_m1 (IDO2, indoleamine 2,3-dioxygenase 2); Rn00574499_m1 (TDO2, tryptophan 2,3-dioxygenase); Rn01411937_m1 (KMO); Rn01449532_m1 (KYNU, kynureninase); and Rn01469327_m1 (HAOO, 3-hydroxyanthranilate 3,4-dioxygenase). The amplification efficiency for each assay (between 1.7 and 2) was determined by running a standard dilution curve. The cycle threshold values were calculated automatically by a CFX Manager v.2.1 software according to the default parameters. RNA abundance was calculated as 2-(threshold cycle). Because the HPRT transcript levels do not significantly change in rats exposed to CCI (Korostynski et al., 2006; Mika, 2008), they served as an adequate housekeeping gene.

### Microglial Cell Cultures

Primary cultures of microglia were prepared from 1-day-old Wistar rat pups as previously described by Zawadzka and Kaminska (2005). The cells were briefly isolated from the rat cerebral cortices, plated at a density of 3 × 10^5^ cells/cm^2^ in culture medium consisting of high-glucose Glutamax DMEM (Gibco, United States) supplemented with heat-inactivated 10% fetal bovine serum (Gibco, United States), 100 U/ml penicillin, and 0.1 mg/ml streptomycin (Gibco, United States) on poly-L-lysine (1 mg/ml; Po282 Sigma)-coated 75-cm^2^ culture flasks and maintained at 37°C in 5% CO_2_. The culture medium was replaced after 4 days. The loosely adherent microglia were recovered after 9 days via mild shaking (80 rpm for 1 h and 100 rpm for 15 min), centrifugation and cell viability was determined via the trypan blue exclusion method using a TC20-automated cell counter (Bio-Rad, Poland). The microglia were suspended in culture medium and plated at a final density of 2 × 10^5^ cells on 24-well plates for mRNA analysis and 6-well plates for immunocytochemistry analysis. The adherent cells were incubated for 48 h in culture medium before analysis. The primary microglial cultures were treated with lipopolysaccharide (LPS, 100 ng/ml, Sigma-Aldrich, United States) for 24 h. Furthermore, to identify the microglia cells in cultures, we used ionized calcium-binding adaptor molecule 1 (IBA1, SC-327225, Santa Cruz Biotechnology Inc., United States; more than 95% of the cells were positive for IBA1). The homogeneity of our cultures was similar to the one obtained by Zawadzka and Kaminska (2005).

### Immunocytochemistry

Morphological changes in microglia were determined via immunocytochemistry using commercially available antibodies against IBA-1. Unstimulated and LPS-stimulated (for 24 h) microglia were cultured on sterile cover slips in 6-well plates (1 × 10^6^ cells/well). The cells were fixed for 20 min in 4% paraformaldehyde (Sigma-Aldrich, St. Louis, MO, United States). Then, the cells were permeabilized with 0.1% Triton^TM^ X-100 (Sigma-Aldrich, St. Louis, MO, United States) in PBS for 30 min at room temperature, washed with PBS, and blocked with 5% donkey serum in PBS. The microglial cells were incubated in primary antibodies [mouse anti-IBA-1 (Santa Cruz Biotechnology Inc., United States)], overnight at 4°C. After washing with PBS, the microglia were incubated for 2–3 h in a fluorophore-conjugated secondary antibody: Alexa Fluor 546 donkey anti-mouse (Molecular Probes) diluted 1:500 in 5% normal donkey serum (NDS). Then, the cells were washed with PBS and coverslipped using Aquatex mounting medium (Merck, Darmstadt, Germany). Morphological changes after LPS administration were evaluated by visualizing microglia under the 40× objective of a Zeiss microscope (Zeiss, Germany). Sections without primary antibodies were used as negative controls.

### Data Analysis

*The in vivo results* (**Figures 1A,B, 3A,B, 4**) are presented in grams or seconds. The inter-group differences were analyzed via one-way ANOVA followed by a Bonferroni’s multiple comparison test. The data are presented as the means ± SEM. The differences were calculated vs. the naïve group and/or vs. the V-treated CCI-exposed group. The qRT-PCR analyses (**Figures 1C–P**, 3C–P) are presented as the fold change compared with the control group (naïve rats). The data are presented as the means ± SEM and were calculated for the ipsilateral sides of the spinal cords and DRG of the CCI-exposed rats. The qRT-PCR data represent the normalized averages derived from the threshold cycle obtained from qRT-PCR. The intergroup differences were analyzed via one-way ANOVA followed by a Bonferroni’s multiple comparison test. The differences were calculated vs. the naïve group and vs. the V-treated CCI-exposed group.

**FIGURE 2:**
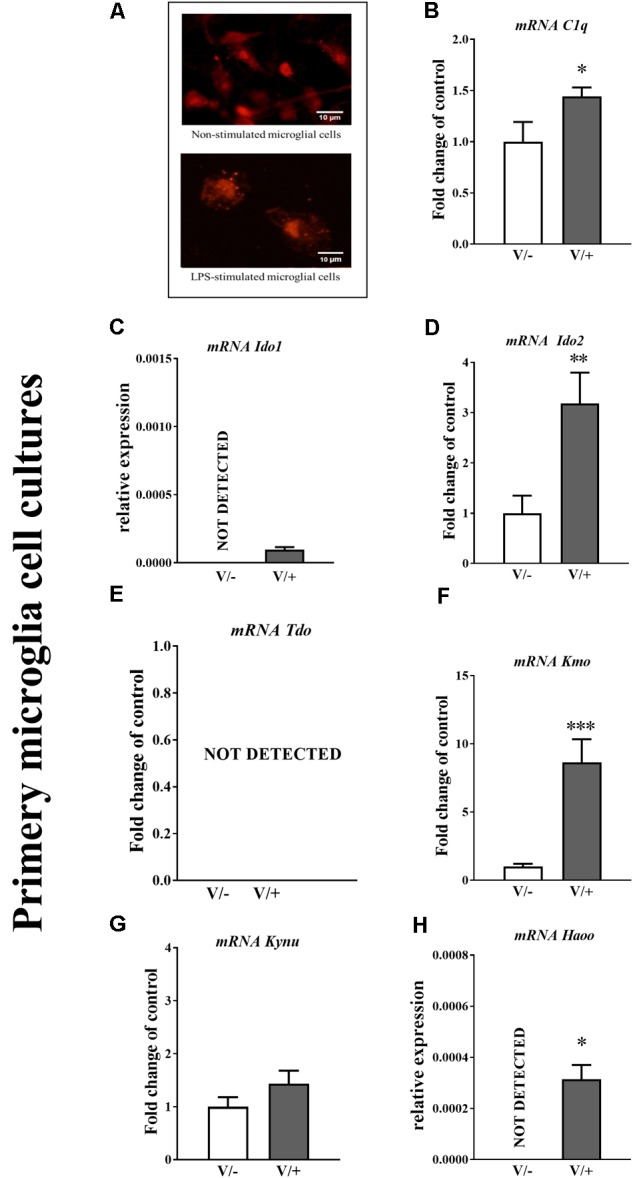
**(A)** Primary microglial cell cultures were treated with vehicle (V) and lipopolysaccharide (LPS; 100 ng/μl). (**B–H)** Immunofluorescence staining for markers of microglia (IBA-1) are presented (scale bar is 10 μm). Enzymes of the kynurenine pathway and microglia marker expression in primary microglial cell cultures. The mRNA levels of *C1q* **(B)**, *IDO1* **(C)**, *IDO2* **(D)**, *TDO* **(E)**, *KMO* **(F)**, *KYNU* **(G)**, and *HAOO* **(H)** in non-stimulated and LPS-treated microglial primary cell cultures. The data are presented as fold change relative to control and relative mRNA level. Inter-group differences were analyzed using a *t*-test. ^∗^*p* < 0.05, ^∗∗^*p* < 0.01, and ^∗∗∗^*p* < 0.001 indicate differences compared to the LPS-treated cells. Abbreviations: V/-, not stimulated; V/+, LPS-stimulated.

*The in vitro results* (**Figure 2**) are presented as the fold change and relative mRNA level and (**Figures 2C,H**) as the mean ± SEM of three independent experiments. Inter-group differences were analyzed with a *t*-test, and the differences were calculated vs. the vehicle-treated non-stimulated primary microglia cells.

## Results

### The Development of Hypersensitivity Appears in Parallel to the Changes in *C1q, IDO1, IDO2, TDO, KMO, KYNU*, and *HAOO* mRNA in the Spinal Cord and DRG 2–28 Days After the CCI

Unilateral, loose ligation of the sciatic nerve caused the development of symptoms typical of neuropathic pain, such as mechanical (**Figure 1A**) and thermal (**Figure 1B**) hypersensitivity. The responses to mechanical stimuli were measured on days 2, 7, 14, and 28 after the CCI using the von Frey test. At all examined time points, these responses were very intense, and all rats exhibited strong hypersensitivity. Compared with naïve rats (24.9 g ± 0.6), strong tactile hypersensitivity had already appeared by day 2 (11.8 ± 0.8 g) and lasted until day 28 (14.7 ± 0.8 g) (**Figure 1A**). Additionally, pronounced cold hypersensitivity was detected on days 2, 7, 14, and 28 after the CCI based on the cold plate test. Compared with naïve rats (27.7 ± 0.7 s), strong thermal hypersensitivity had already appeared by day 2 (9.8 ± 0.7 s) and lasted until day 28 (19.8 ± 0.9 s) (**Figure 1B**).

The qRT-PCR analysis showed that the *C1q* mRNA level in the spinal cord was elevated after the CCI at all examined time points. The changes in *C1q* mRNA expression found in the spinal cord on day 2 (3.6-fold, *p* < 0.001) became more severe on day 7 (9.6-fold, *p* < 0.001), while around day 14, they gradually decreased (6.9-fold, *p* < 0.001), although the changes remained significant until day 28 (5.1-fold, *p* < 0.001) (**Figure 1C**). In the DRG, the upregulation of *C1q* mRNA was detected on days 2, 7, 14, and 28 (2.2-, 3.8-, 4.4-, and 2.9-fold, respectively; *p* < 0.001) (**Figure 1D**). The *IDO1* mRNA was not detectable in the spinal cord (**Figure 1E**). Significant changes in *IDO1* mRNA expression were found in the DRG on days 7, 14, and 28 (3.7-, 4.5-, and 3.7-fold, respectively; *p* < 0.001) (**Figure 1F**). The *IDO2* mRNA (1.0 ± 0.15 vs.1.7 ± 0.2) was upregulated 1.7-fold, *p* < 0.01 compared with that of naïve rats only on day 7 after the CCI in the spinal cord (**Figure 1G**). In the DRG, the upregulation of *IDO2* mRNA was detected on days 7 and 14 (2.1-fold, *p* < 0.05 and 2.9-fold, p < 0.001, respectively) (**Figure 1H**). In the spinal cord, *TDO* mRNA was not changed after the CCI at all examined time points (**Figure 1I**). In the DRG, the downregulation of *IDO2* mRNA was detected on days 2 and 7 (1.5-fold, *p* < 0.001 and 1.4-fold, *p* < 0.001, respectively) (**Figure 1J**). The *KMO* mRNA level in the spinal cord was elevated after the CCI on days 2, 7, and 14 by 3.0-fold, *p* < 0.001; 2.8-fold, *p* < 0.05; and 2.7-fold, *p* < 0.01, respectively (**Figure 1K**). In the DRG, the *KMO* mRNA level was upregulated on days 7 and 14 after the CCI (3.3-fold, *p* < 0.001 and 2.1-fold, *p* < 0.05, respectively) (**Figure 1L**). In both examined structures, we did not detect any changes in *KYNU* mRNA expression (**Figures 1M,N**). The *HAOO* mRNA level in the spinal cord was elevated after the CCI at all examined time points. The following changes in *HAOO* mRNA expression were found in the spinal cord on days 2, 7, 14, and 28: 2.7-fold, *p* < 0.001; 2.2-fold, *p* < 0.001; 2.5-fold, *p* < 0.001; and 1.7-fold, *p* < 0.01, respectively (**Figure 1O**). In the DRG, the upregulation of *HAOO* mRNA was also detected at all examined time points – days 2, 7, 14, and 28 (2.2-fold, *p* < 0.01; 2.6-fold, *p* < 0.001; 4.6-fold, *p* < 0.001; and 2.7-fold, *p* < 0.001, respectively) (**Figure 1P**).

### The Changes in *C1q, IDO1, IDO2, TDO, KMO, KYNU*, and *HAOO* mRNA in Primary Microglial Cell Cultures After the LPS Stimulation

Our immunocytochemical analyses (**Figure 2A**) demonstrated that the morphology of microglia was altered from a ramified (V/-, vehicle-treated non-stimulated) to an amoeboid appearance after the treatment with LPS for 24 h. The qRT-PCR analysis showed basal levels of *C1q, IDO2, KMO*, and *KYNU* mRNA in primary microglial cell cultures (**Figures 2B,D,F,G**). The LPS treatment significantly enhanced the levels of *C1q, IDO1, IDO2, KMO*, and *HAOO* mRNA as measured after 24 h (**Figures 2B,C,D,F,H**), but the mRNA for *TDO* was still undetectable (**Figure 2E**).

### The Influence of the Repeated Administration of Minocycline on the Development and on *C1q, IDO1, IDO2, TDO, KMO, KYNU*, and *HAOO* mRNA in the Spinal Cord and DRG 7 Days After the CCI

**FIGURE 3:**
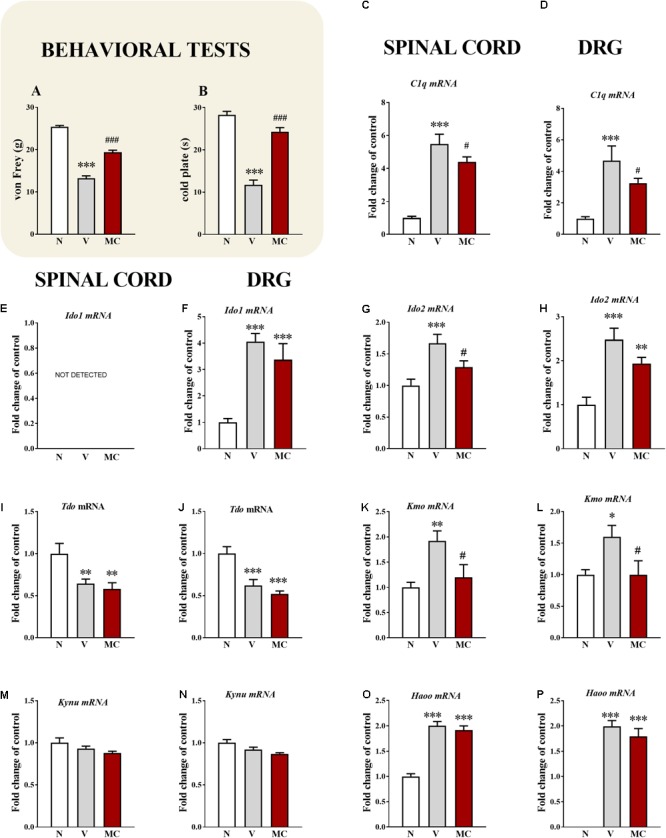
**(A,B)** Influence of the pre-emptive and repeated administration of minocycline (30 mg/kg; *i.p*.; 16 and 1 h before the CCI and then for 7 days twice daily) on the development of mechanical (**A**; von Frey test) and thermal (**B**; cold plate test) hypersensitivity 7 days after the CCI in rats. The data are presented as the mean ± SEM (10–20 rat per group). **(C–P)** Effects of minocycline (**M**; 30 mg/kg; *i.p*.; 16 and 1 h before the CCI and then twice daily for 7 days) on the mRNA levels of *C1q* **(C,D)**, *IDO1* **(E,F)**, *IDO2* **(G,H)**, *TDO* **(I,J)**, *KMO* **(K,L)**, *KYNU* **(M,N)**, and *HAOO* **(O,P)** in the spinal cord and DRG during neuropathic pain and minocycline treatment. Behavioral tests were assessed 60 min after the drug administration. Biochemical results were analyzed as fold changes compared to controls and represent the normalized averages derived from the qRT-PCR threshold cycles of 5–12 samples from each group. The data are presented as the mean ± SEM. Inter-group differences were analyzed using a Bonferroni’s multiple comparison test. ^∗^*p* < 0.05, ^∗∗^*p* < 0.01, and ^∗∗∗^*p* < 0.001 indicate differences compared to the naïve rats. ^#^*p* < 0.05, and ^###^*p* < 0.001 indicate differences compared to the CCI-exposed group. N, naïve; V, vehicle (water for injection); MC, minocycline.

In the von Frey test, strong tactile hypersensitivity on the paw ipsilateral to the injury was observed on day 7 after the CCI. At this time, the ipsilateral paw responded to a stimulation of 13.2 ± 0.6 g (**Figure 3A**), compared to the reaction of the hind paws in the naïve rats to 25.4 ± 0.3 g. The strongest thermal hypersensitivity was observed on the 7th day in the cold plate test (**Figure 1B**). At this time, the ipsilateral paw reacted after 11.7 ± 1.1 s (**Figure 3B**), compared to the reaction after 28.3 ± 0.8 s in the naïve rats. The administration of minocycline caused a significant reduction in the above symptoms. Pre-emptive and repeated treatment with minocycline (twice daily; 30 mg/kg *i.p.*) significantly attenuated the mechanical hypersensitivity to 19.4 ± 0.5 g (**Figure 3A**) and thermal hypersensitivity to 24.3 ± 1.0 s (**Figure 3B**) on day 7 after the CCI.

The qRT-PCR analysis showed that at the spinal cord level, *C1q* mRNA was upregulated 5.4-fold (*p* < 0.001), compared to that of the naïve rats (**Figure 3C**). Minocycline diminished the level of *C1q* mRNA in the spinal cord 1.25-fold (*p* < 0.05). Additionally, in the DRG, *C1q* mRNA was significantly upregulated 4.7-fold (**Figure 3D**) in the CCI-exposed rats compared to naïve animals. Minocycline significantly diminished the level of *C1q* in the DRG 1.4-fold (*p* < 0.05) (**Figure 1D**). The IDO1 mRNA was also not detectable in the spinal cord (**Figure 3E**) but was upregulated in the DRG (4.1-fold, **Figure 3F**) in the CCI-exposed rats compared with naïve animals. Repeated treatment with minocycline did not influence the level of *IDO1* in the DRG (**Figure 3F**). Spinal *IDO2* mRNA was upregulated 1.7-fold compared to that of naïve rats (**Figure 3G**). Minocycline diminished (1.3-fold, *p* < 0.05) the level of *IDO2* mRNA in the spinal cord. Additionally, in the DRG, *IDO2* mRNA was significantly upregulated 2.5-fold (**Figure 3H**) in the CCI-exposed rats compared to the naïve animals. Minocycline induced changes in the level of *IDO2* in the DRG from 2.5- to 1.9-fold (**Figure 3H**). In both examined structures, *TDO* mRNA was downregulated 1.4-fold in the CCI-exposed rats compared to the naïve animals (**Figure 3I**), and minocycline did not influence its levels. In the spinal cord, *KMO* mRNA was upregulated 1.9-fold compared to that of the naïve rats (**Figure 3K**). Minocycline strongly diminished the level of *KMO* mRNA in the spinal cord 1.7-fold (*p* < 0.05). Additionally, in the DRG, *KMO* mRNA was significantly upregulated 1.6-fold (**Figure 3L**) in the CCI-exposed rats compared to the naïve animals. Minocycline significantly diminished the level of *KMO* in the DRG 1.6-fold (*p* < 0.05) (**Figure 1L**). In the spinal cord and in the DRG, we did not observe any changes in the *KYNU* mRNA level in the CCI-exposed rats after minocycline treatment (**Figures 3M,N**). In both examined structures, *HAOO* mRNA was upregulated twofold (spinal cord) and 1.9-fold (DRG) in the CCI-exposed rats compared to the naïve animals (**Figures 3O,P**). However, minocycline did not influence the level of *HAOO* in the spinal cord and DRG (**Figures 3O,P**).

### The Effect of 1-D-MT and UPF 648 *i.t.* Administration on the Development of Mechanical and Thermal Hypersensitivity in the CCI-Exposed Rats

**FIGURE 4:**
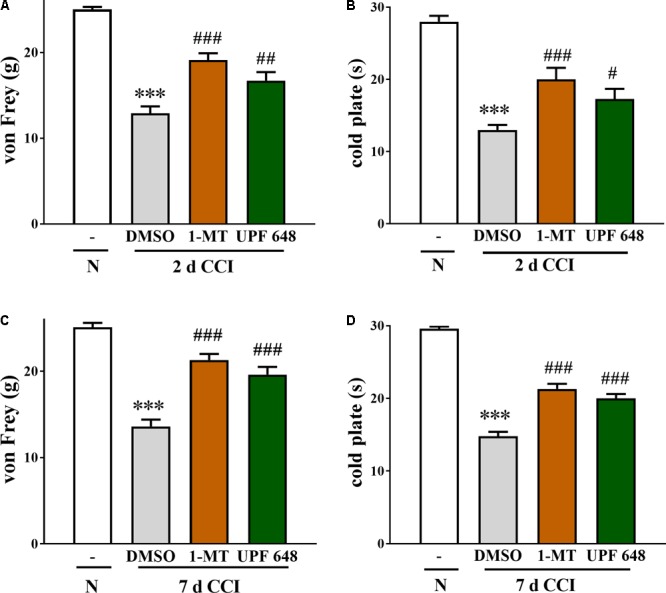
The effect of the *i.t.* administration of an IDO2 inhibitor (1-MT) and a KMO inhibitor (UPF 648) at a dose of 20 μg/5 μl on mechanical (von Frey test; **A,C**) and thermal (cold plate test; **B,D**) hypersensitivity in rats on days 2 and 7 after the CCI. Both KMO inhibitors significantly reduced hypersensitivity. The results are presented as the means ± SEM (10–16 rats per group). Inter-group differences were analyzed via one-way ANOVA followed by a Bonferroni’s test for multiple comparisons. ^∗∗∗^*p* < 0.001 indicates a difference compared to the naïve group; ^#^*p* < 0.05, ^##^*p* < 0.01, and ^###^*p* < 0.001 indicate differences compared to the V-treated CCI-exposed group. N, naïve group; V, vehicle (DMSO-treated CCI exposed group); 1-D-MT, IDO2 inhibitor; UPF 648, KMO inhibitor.

The effect of the repeated once-a-day i.t. administration of IDO2 (1-D-MT) and KMO (UPF 648) inhibitors (both at a dose of 20 μg) was studied on days 2 (**Figures 4A,B**) and **7** (**Figures 4C,D**) in the CCI-exposed rats.

In the von Frey test on day 2 after the CCI, the paw ipsilateral to the injury significantly responded to the stimulation of 12.9 ± 0.8 g in the vehicle-treated CCI-exposed rats compared to the reactions of the hind paws of naïve rats to 25.0 ± 0.3 g (**Figure 4A**). Pre-emptive and repeated treatment with 1-D-MT and UPF 648 significantly attenuated the mechanical hypersensitivity to 19.1 ± 0.8 and 16.7 ± 0.1 g, respectively (**Figure 4A**). On day 2 after the CCI, the strongest thermal hypersensitivity was observed; the ipsilateral paw of the CCI-exposed rats reacted after 13.0 ± 0.7 s (**Figure 4B**) compared to the reaction after 28.0 ± 0.8 s in the naïve rats. Pre-emptive and repeated treatment with 1-D-MT and UPF 648 significantly attenuated the thermal hypersensitivity development to 20.0 ± 1.6 and 17.3 ± 1.4 s, respectively (**Figure 4B**).

In the von Frey test on day 7 after the CCI, similar to day 2, the paw ipsilateral to the injury responded to the stimulation in the vehicle-treated CCI-exposed rats of 13.6 ± 0.8 g compared to the reactions of the hind paws in the naïve rats to 25.1 ± 0.5 g (**Figure 4C**). Pre-emptive and repeated treatment with 1-D-MT and UPF 648 significantly attenuated the mechanical hypersensitivity to 21.3 ± 0.7 and 19.6 ± 0.9 g, respectively (**Figure 4C**). On day 7 after the CCI, similar to day 2, strong thermal hypersensitivity was observed, and the ipsilateral paw reacted after 14.8 ± 0.3 s (**Figure 4B**) compared to the reaction after 29.6 ± 0.3 s in the naïve rats. Pre-emptive and repeated treatment with 1-D-MT and UPF 648 significantly attenuated the thermal hypersensitivity development to 21.3 ± 0.7 and 20.0 ± 0.6 s, respectively (**Figure 4D**).

## Discussion

The kynurenine pathway has received increasing attention as its connection to neurodegenerative conditions became more apparent. Here, we present the collection of the first evidence revealed in a single study on the potential contributions to neuropathic pain development of the enzymes of the kynurenine pathway at the spinal cord and DRG levels. At the spinal cord level, *IDO2, KMO*, and *HAOO* mRNA levels were elevated as measured on day 7 after the CCI. Our results suggest that the influx and/or activation of C1q-positive cells, e.g., microglia and macrophages, is responsible for these changes. According to our data obtained from primary glial cell cultures, all enzymes of the kynurenine pathway, except TDO, are derived from microglial cells. The higher upregulation of *IDO2* and *KMO* mRNA levels was observed 24 h after the LPS stimulation. Our pharmacological studies give evidence that the repeated intraperitoneal administration of minocycline, a microglia/macrophage inhibitor, not only attenuated tactile and thermal hypersensitivity but also diminished the levels of *IDO2* and *KMO* mRNA. Our further pharmacological studies confirm that IDO2 and KMO enzymes take part in the development of neuropathic pain, since their inhibitors significantly diminish hypersensitivity development after nerve injury.

The kynurenine pathway starts with a rapid conversion of L-tryptophan to L-kynurenine, and then to kynurenine. The metabolism of L-tryptophan occurs under the influence of three enzymes: TDO and IDO1 and 2. It has been shown that the TDO level does not change or even decrease in pathological conditions (Comai et al., 2005; Braidy et al., 2011). These results are consistent with our observations where we demonstrated that the level of *TDO* mRNA decreases on days 2 and 7 after CCI in the DRG, while in the spinal cord, there are no significant changes. Two enzymes, IDO1 and 2, attracted our attention because, as is evident from the published data, they are important in the development of neurodegenerative diseases, however, their role in neuropathic pain has not been established.

The conversion of kynurenine to 3-hydroxykynurenine by KMO, which is further metabolized through KYNU to 3-hydroxyanthranilic acid, is then metabolized by HAOO to an unstable compound that is enzymatically transformed into picolinic acid and non-enzymatically converted to quinolinic acid. The subsequent products of the kynurenine pathway are predominantly synthesized by infiltrating macrophages and microglia (Chen and Guillemin, 2009). There is a rapidly growing body of evidence indicating that microglia/macrophages have causal roles in the pathogenesis of pain hypersensitivity following nerve injury (Rojewska et al., 2014c; Popiolek-Barczyk and Mika, 2016). Importantly, for neuropathy, in parallel with the influx and/or activation of C1q-positive cells, we observed an increase in the levels of mRNA of enzymes of the kynurenine pathway, which suggests its role in the development of pain. In the present study, we have observed that after nerve injury, *IDO1* and *IDO2* mRNA levels were increased in the DRG on days 7 and 14. In the spinal cord, *IDO1* mRNA was not detectable; however, an increase in the *IDO2* mRNA level was observed on day 7 after the CCI. According to the literature (Jones et al., 2015; Mbongue et al., 2015) and our data obtained from primary glial cell cultures, IDO1 and IDO2 enzymes are derived from microglia and macrophages cells; however, the level of IDO2, in particular, is enhanced. Data from the literature indicate that in many pathologic states (cerebral ischemia, Alzheimer’s disease, Parkinson’s disease, Huntington’s disease, multiple sclerosis, amyotrophic lateral sclerosis, autoimmune diseases, and tumor), IDO1/2 activity increases in microglial/macrophage cells (Munn, 1998; Widner et al., 1999; Tikka et al., 2001; Muller et al., 2005; Stoy et al., 2005; Munn and Mellor, 2007; Darlington et al., 2007; Opitz et al., 2007; Zelante et al., 2009; Laugeray et al., 2010; Sage et al., 2014; Mazarei and Leavitt, 2015). Basing on our results, we hypothesized that the suppression of spinal IDO2 would diminish the kynurenine pathway activation and as a consequence, the development of neuropathic pain. Therefore, we chose an inhibitor of IDO2: 1-D-MT for further research (Novak et al., 1995; Metz et al., 2007). Our team for the first time have shown that the inhibition of IDO2 activity is important in the development of hypersensitivity after sciatic nerve injury. The metabolism of L-tryptophan occurs under the influence of three enzymes: TDO and IDO1 and 2; however, in neuropathic pain, the key enzyme is IDO2. Importantly, IDO inhibitors have been suggested in basic cancer studies as novel and therapeutic (Muller et al., 2005) and recently, in studies on human cancer cells 1-D-MT has been confirmed to have beneficial properties (Opitz et al., 2011), the possibility of its use in the treatment of neuropathic pain needs to be studied.

Another important enzyme of the kynurenine pathway is KMO, which converts kynurenine to 3-hydroxy-L-kynurenine. KMO biases the balance toward the more extensive production of neuroexcitotoxic quinolinic acid and toward the lower production of neuroprotective kynurenic acid. Thus, decreasing the activity of KMO may be one of the ways to protect neurons from excitotoxicity (Carpenedo et al., 1994). The increased level of KMO leads to the formation of 3-hydroxy-L-kynurenine, which, when administered to the ventricles of the brain, causes convulsions (Lapin, 1981) and neuronal damage (Novak et al., 1995; Nakagami et al., 1996). Neurotoxicity from 3-hydroxy-L-kynurenine, associated with the generation of active hydroperoxide radicals (Coyle and Puttfarcken, 1993), is observed in many neurodegenerative diseases (Pearson and Reynolds, 1992; Sardar et al., 1995). It has been shown that KMO inhibitors (Ro61-804 and its prodrug JM6) are neuroprotective in ischemia (Röver et al., 1997; Moroni et al., 2005), dyskinesia (Grégoire et al., 2008), Alzheimer’s disease (Chin et al., 2005), and Huntington’s disease (Zwilling et al., 2011) and that they are able to diminish the activation of microglia and, as a consequence, the production of cytokines (Croitoru-Lamoury et al., 2003; Chin et al., 2005). We have also shown that the inhibition of KMO by Ro61-8048 and its prodrug JM6 was analgesic under neuropathic pain conditions (Rojewska et al., 2016). Recently, a new inhibitor referred to as UPF 648 has been created, and it behaves as a more potent and selective KMO inhibitor and is structurally unrelated to Ro-618048 (Ceresoli-Borroni et al., 2007; Amaral et al., 2013). The study conducted by Amori et al. (2009) demonstrated that UPF 648 administered after an intrastriatal quinolinic acid injection not only decreased both neurotoxic 3-hydroxy-L-kynurenine and quinolinic acid production (by 77 and 66%, respectively) but also moderately raised neuroprotective kynurenic acid synthesis (by 27%). We have obtained the first results showing that under neuropathy, UPF 648 reduces the hypersensitivity induced by sciatic nerve injury. The newly tested KMO inhibitor caused analgesia similarly to that observed by JM6 and Ro61-8048 administration (Rojewska et al., 2016). Our data once again suggest KMO as an important target for neuropathy treatment.

The next step of the kynurenine pathway is the conversion of 3-hydroxy-L-kynurenine into 3-hydroxyanthranilic acid by KYNU. Our study on primary microglial cell cultures indicated that the KYNU mRNA level is low and unchanged after LPS stimulation. According to our present study, the mRNA level of KYNU was not changed in the spinal cord as well as in the DRG after sciatic nerve ligation at any points examined.

The next step of the kynurenine pathway is the conversion of 3-hydroxyanthranilic acid into the very neurotoxic product quinolinic acid through HAOO. Our results indicated that the HAOO mRNA level is upregulated at the spinal cord and DRG levels. The research conducted on primary microglial cell cultures has confirmed the increase in HAOO mRNA levels 24 h after LPS stimulation, but it was not as strong as the one observed in case of IDO2 and KMO. Elevated activity of HAOO has been observed in ischemia (Saito et al., 1993) and Huntington’s disease (Schwarcz et al., 1988), and as a consequence, quinolinic acid has been shown to be increased in autoimmune and neurodegenerative diseases (e.g., cerebral ischemia, Alzheimer’s disease, Huntington’s disease, and multiple sclerosis) (Flanagan et al., 1995; Widner et al., 1999; Chiarugi et al., 2001; Guidetti et al., 2004). Recently, numerous studies have highlighted the role of macrophages and microglial cells in this phenomenon (Schwarcz et al., 2012).

In our previously published study (Rojewska et al., 2014a) using microarray methods, we selected genes that are modulated after minocycline administration. Our attention was drawn to an enzyme of the kynurenine pathway, KMO. Minocycline is known as an inhibitor of microglia/macrophage activation with strong analgesic properties under neuropathic pain (Raghavendra et al., 2003; Ledeboer et al., 2005; Mika et al., 2007, 2010; Rojewska et al., 2014a,b,c). Ryu et al. (2006) showed that minocycline reduces the quinolinic acid-evoked microglia activation. Ryu et al. (2006), we showed that minocycline in primary microglial cell culture significantly reduces KMO mRNA levels after LPS stimulation. Our present *in vitro* results give evidence that microglial cells are a cellular source of all enzymes of the kynurenine pathway except TDO. However, the strongest upregulation which could be observed in *in vivo* and *in vitro* studies occurred in the IDO2 and KMO levels. Our *in vivo* study confirmed that the repeated *i.p.* administration of minocycline significantly decreases the IDO2 and KMO mRNA levels in the spinal cord and in the DRG. Therefore, for further pharmacological studies, we chose two inhibitors of kynurenine pathway enzymes. The selected inhibitors, 1-D-MT (for IDO2) and UPF 648 (for KMO), strongly reduce the development of hypersensitivity to mechanical and thermal stimuli in a rat neuropathic pain model. The growing body of evidence suggests that drugs, which are able to influence directly [1-D-MT, UPF 648, Ro61-8048 (Rojewska et al., 2016)] or indirectly [minocycline (Rojewska et al., 2014a) aspirin (Schroecksnadel et al., 2005)] IDO and/or KMO have anti-inflammatory properties. Direct translation to the human situation is not possible, since tryptophan metabolism in rats is different from its metabolism in humans (Leklem, 1971). However, our results suggest that the inhibition of the IDO2 and KMO enzymes is very important in the activation of the kynurenic pathway, and therefore, its therapeutic significance needs to be studied.

## Conclusion

Recent studies highlight the involvement of the kynurenine pathway in the pathology of neurodegenerative diseases, but the role of this system in neuropathic pain requires further extensive research. The results of our studies show that the IDO2 and KMO enzymes of the kynurenine pathway are important for the development of neuropathic pain and indicate that they represent a novel pharmacological target for the treatment of neuropathy. We have observed that the inhibition of IDO2 and KMO may decrease the negative effects of nerve damage and consequently improve neuropathic pain therapy. Our results are supported by preclinical evidence that shows that the administration of KMO inhibitors counteracted the effects of neuroinflammation in various neurodegenerative diseases (Campbell et al., 2014; Wilson et al., 2014). In our opinion, IDO2 is a new and important target for neuropathy treatment, nevertheless, this requires further in-depth research. Interestingly, it also appears that the pharmacological indirect modulation of the kynurenine pathway by microglia/macrophages inhibitors might provide satisfactory therapeutic effects in the future. Our research and others’ data encourage further studies and the search for new-generation drugs able to modify the kynurenine pathway, especially for their use in neuropathic pain treatment.

## Author Contributions

ER and JM supervised the project and designed the detailed experiments and wrote or contributed to the writing and revision of the manuscript. ER, KC, AP, WM, and JM performed the study, and collected and analyzed the data. All authors commented on the study and approved the final manuscript.

## Conflict of Interest Statement

The authors declare that the research was conducted in the absence of any commercial or financial relationships that could be construed as a potential conflict of interest.
